# Supplementary material for: Mapping the evolution of fertility support policies in China: A content and instrumental analysis
Source: PLoS One. 2025 Oct 9;20(10):e0332137. doi: 10.1371/journal.pone.0332137 (PMC12510515; doi:10.1371/journal.pone.0332137)
Supplement: S1 Appendix — (ZIP) [file pone.0332137.s001.zip › S1 Appendix. 226 original policy documents/19-国家卫生和计划生育委员会关于加强计划生育基层基础工作的指导意见(FBM-CLI-4-229198).doc]

国家卫生和计划生育委员会关于加强计划生育基层基础工作的指导意见

**发布部门：** [国家卫生和计划生育委员会(已撤销)](https://www.pkulaw.com/law/chl?Aggs.IssueDepartment=60319) [机构沿革](javascript:void(0))

**发文字号：**国卫指导发〔2014〕37号

**发布日期：**2014.07.02

**实施日期：**2014.07.02

**时效性：** [现行有效](https://www.pkulaw.com/law/chl?Aggs.TimelinessDic=01)

**效力级别：** [部门工作文件](https://www.pkulaw.com/law/chl?Aggs.EffectivenessDic=XE0303)

**法规类别：** [计划生育管理](https://www.pkulaw.com/law/chl?Aggs.Category=09705)

国家卫生和计划生育委员会关于加强计划生育基层基础工作的指导意见
（国卫指导发〔2014〕37号）

各省、自治区、直辖市卫生计生委（人口计生委），新疆生产建设兵团人口计生委：
　　为贯彻落实党的十八大和十八届二中、三中全会精神以及《中共中央 国务院关于调整完善生育政策的意见》（中发〔2013〕15号，以下简称中央《意见》），坚持计划生育基本国策，做好机构改革和调整完善生育政策工作，促进人口长期均衡发展，现就加强计划生育基层基础工作提出以下意见。
　　**一、**充分认识加强基层基础工作的重要意义
　　实行计划生育以来，在党中央、国务院的正确领导下，各地坚持党政一把手亲自抓、负总责，坚持实行计划生育目标管理责任制和“一票否决”制，重视和加强基层基础工作，贯彻宣传教育为主、避孕为主、经常性工作为主的方针，形成了行政管理、技术服务、群众工作“三位一体”的工作网络，建立了顾全大局、扎根基层、求真务实、无私奉献的工作队伍。经过40多年的艰苦努力，计划生育工作取得了巨大成就，为促进经济社会持续健康发展、全面建成小康社会创造了有利的人口环境。
　　进入新世纪，我国人口形势更加复杂，人口众多仍然是长期面临的基本国情，人口结构性问题日益成为影响经济社会发展的重要因素。党的十八大明确提出，坚持计划生育的基本国策，提高出生人口素质，逐步完善政策，促进人口长期均衡发展。党的十八届二中全会决定整合卫生和计划生育部门，十八届三中全会决定启动实施单独两孩政策，中央《意见》对全面做好新时期计划生育工作进行了部署。落实好中央的重大决策，必须全面加强计划生育基层基础工作。这是实现计划生育工作转型发展、更好地为群众服务、让群众满意的迫切需要；是加快推进机构改革、转变政府职能的客观要求；是稳妥扎实有序落实单独两孩政策、稳定适度低生育水平、促进人口长期均衡发展的根本保障。各地要提高认识，改革创新，切实加强计划生育基层基础工作，努力开创事业发展的新局面。

　　**二、**基本原则和工作目标
　　（一）基本原则。
　　一是坚持以人为本。尊重群众计划生育主体地位，寓管理于服务之中，切实维护群众实行计划生育的合法权益，提高人民群众的满意度。
　　二是坚持重心下移。稳定和健全基层工作网络和队伍，完善制度，加大投入，加强培训，提高待遇，保证基层有人管事、有钱办事、照章理事。
　　三是坚持资源整合。充分发挥基层卫生和计划生育的优势，促进资源整合、信息共享，加强与相关部门沟通协调，形成齐抓共管、综合治理的格局。
　　四是坚持改革创新。更加注重人文关怀，更加注重利益导向，更加注重宣传引导，全面推进依法行政，改革基层服务管理模式，实现工作思路和工作方法的根本转变。
　　（二）工作目标。
　　当前和今后一个时期，以全面深化改革为动力，协调推进计划生育体制机制、政策制度、方式方法的改革，健全管理科学、服务优质、运行规范的计划生育工作网络，建设结构合理、素质优良、保障有力的计划生育基层工作队伍，完善宣传教育、依法管理、群众自治、优质服务、政策推动、综合治理的基层工作长效机制，加快推进计划生育治理体系和治理能力现代化，全面提高基层计划生育服务管理水平，为落实计划生育基本国策、逐步调整完善生育政策、促进人口长期均衡发展奠定坚实的基础，提供有力的保障。

　　**三、**切实巩固和加强基层工作网络
　　（一）稳定行政管理机构和队伍。在卫生计生机构改革中，确保基层计划生育工作只能加强，不能削弱。县级卫生和计划生育部门要合理设置相关机构，充实力量，明确职责，履行好计划生育服务管理职能。健全乡镇（街道）计划生育办公室，按常住人口比例合理配备工作人员，承担宣传计划生育法律法规、落实奖励优待政策、统计婚育和出生信息、指导开展技术服务、查处违法生育等任务，督促辖区内机关、企事业单位、社会团体落实计划生育责任，指导村（居）民委员会做好计划生育工作，履行基层公共卫生、卫生监督等职能。
　　（二）优化配置服务机构和队伍。认真贯彻国家卫生计生委、中央编办《关于优化整合妇幼保健和计划生育技术服务资源的指导意见》（国卫妇幼发〔2013〕44号），有效整合县、乡妇幼保健和计划生育技术服务资源，优势互补，增强基层计划生育技术服务能力，保障计划生育宣传教育、技术服务、优生指导、药具发放、信息咨询、随访服务、生殖保健、人员培训等职责的落实。
　　（三）加强村（居）计划生育队伍建设。村（居）党组织、村（居）民委员会要切实承担计划生育工作职责，健全计划生育领导小组或计划生育委员会，配强配齐专职工作人员，合理确定工资、福利待遇。村（居）计划生育专职工作人员经过培训可以兼任新农合监督、疾病防控、卫生监督信息员。机关、企事业单位、社会团体要配备计划生育专（兼）职工作人员。
　　（四）健全完善基层计划生育协会组织。充分发挥计生协生力军作用，加强领导班子建设，健全基层工作网络。通过政策引导、项目支持等方式，大力支持协会发展，增强协会的活力和能力。在新经济组织、新社会组织和流动人口集聚地普遍建立计划生育协会。

　　**四、**扎实做好基层经常性工作
　　（一）加强宣传引导。通过多种形式和渠道，宣传我国人口国情、计划生育基本国策和计划生育工作取得的巨大成就，增强全社会的国情、国策意识；宣传计划生育法律法规、调整完善生育政策措施，促进人口长期均衡发展；大力推进“婚育新风进万家”活动，宣传避孕节育、优生优育、生殖健康知识，倡导科学、文明、进步的婚育观念。做好信息公开和政策解读，回应社会关切。
　　（二）加强依法管理。按照合法行政、合理行政、程序正当、高效便民、诚实守信、权责统一的要求，改进执法方式，推进基层依法管理规范化建设。规范社会抚养费征收管理，严格执行“收支两条线”。积极推进生育服务证制度改革，简化再生育审批。加大流动人口计划生育服务管理，推进流动人口卫生和计划生育基本公共服务均等化。深入开展“关爱女孩行动”，依法查处“两非”案件，综合治理出生人口性别比偏高问题。深入开展阳光计生行动，做好信访维稳工作。
　　（三）加强技术服务。开展计划生育、优生优育、生殖健康服务，推进避孕方法知情选择，引导群众选择安全、适宜、长效为主的避孕措施。严格禁止非医学需要的大月份引产。全面实施免费孕前优生健康检查项目，开展出生缺陷综合防治，提高出生人口素质。落实国家免费计划生育基本技术服务项目，严格服务规程，确保服务质量。
　　（四）加强利益导向。落实法定奖励政策，实施农村部分计划生育家庭奖励扶助制度、计划生育家庭特别扶助制度和西部地区“少生快富”工程等“三项制度”。做好计划生育特殊困难家庭的经济扶助、养老保障、医疗保障、社会关怀等工作。鼓励社会力量为计划生育家庭提供服务。全面开展创建幸福家庭活动、新家庭计划和生育关怀行动。
　　（五）加强信息管理。依托村（居）工作网络和队伍，落实定期采集信息制度。协调建立孕期保健、住院分娩、免疫规划、户籍管理、迁移流动、婚姻登记、义务教育、社会保障等信息共享机制，做好出生人口统计与监测工作。深入开发利用全员人口信息资源，运用信息化手段，精简基层台账、报表。提高信息质量，保障信息安全。
　　（六）加强群众自治。充分发扬民主，依法制订计划生育群众自治章程和计划生育公约，明确村（居）民委员会的计划生育职责、计划生育协会的任务、群众应享有的权利和义务，结合实际制订计划生育奖励、制约措施，引导群众自我管理、自我服务、自我教育、自我监督。加大诚信计生推进力度，实行政府或村（居）民委员会与群众“双向承诺，互为守信”。

　　**五、**加强组织领导
　　（一）落实领导责任。继续坚持计划生育党政一把手亲自抓、负总责，坚持目标管理责任制考核，坚持“一票否决”制。坚持和完善计划生育领导小组制度，着力研究解决制约基层计划生育发展的突出问题，确保责任到位、措施到位、投入到位、落实到位。
　　（二）加强综合治理。明确各部门计划生育工作职责，加强政策统筹，加强工作协调，加强信息共享，促进相关部门经济社会政策和服务管理措施与计划生育工作有机衔接，形成齐抓共管计划生育工作的政策环境和工作机制。综合治理措施不得与法律法规相抵触。
　　（三）加大投入力度。落实《财政部 [人口计生委关于完善人口和计划生育投入保障机制的意见](https://www.pkulaw.com/chl/7dedc9a68c27375dbdfb.html)》（财教〔2011〕558号）精神，健全稳定增长的财政投入保障机制，确保计划生育财政投入增长幅度要高于经常性财政收入增长幅度，确保法律法规规定的各项奖励扶助和优惠政策、免费基本技术服务、基层机构和队伍建设、经常性工作等经费的落实。不得将计划生育财政投入与社会抚养费征收挂钩。
　　（四）提升队伍能力。研究制订基层计划生育工作队伍建设规划，合理配置人才资源，引进优秀人才，加大教育培训力度，提升学历层次，以能力建设为核心推进队伍职业化建设。强化宗旨意识、法治意识、服务意识，增强做好计划生育工作的责任感和使命感。从政治、经济、生活上关心关爱基层工作者，对先进人物给予表彰和奖励。
　　（五）实行创建引领。深入开展计划生育优质服务先进单位创建活动，坚持改革创新，完善指标体系，改进评估方式，加强过程管理，实行定期复评，建立激励和退出机制，确保计划生育优质服务先进单位创建活动的示范性和导向性，带动整体服务管理水平的提高。
　　（六）改进考核评估。科学设定目标管理责任制考核指标，把党政重视、部门协同、群众参与、队伍建设、经费保障、依法行政、利益导向作为目标考核的重要内容。简化考核程序，减少考核频次，探索建立第三方评估机制。加强对考核结果的运用，严格兑现奖惩。
　　各地要结合实际，认真贯彻落实本意见精神，我委将适时组织督查。

国家卫生计生委
2014年7月2日

© 北大法宝：（[www.pkulaw.com](https://www.pkulaw.com/)) 专业提供法律信息、法学知识和法律软件领域各类解决方案。北大法宝为您提供丰富的参考资料，正式引用法规条文时请与标准文本核对。
欢迎查看所有[产品和服务](https://www.pkulaw.com/helps/2.html)。法宝快讯：[如何快速找到您需要的检索结果？](https://www.pkulaw.com/helps/70.html)[法宝](https://www.pkulaw.com/helps/69.html)V6有何新特色？

扫描二维码阅读原文

**原文链接：**<https://www.pkulaw.com/chl/89ea458535dbdff3bdfb.html>
